# miR-30d-5p inhibits proliferation, invasion and migration of breast cancer cells by targeting SERPINE1 and promoting fatty acid β-oxidation

**DOI:** 10.18632/aging.205587

**Published:** 2024-02-22

**Authors:** Lina Zhang, Shuguang Ren, Yang Sang, Yueyang Hu, Cong Wang, Xinrui Wang, Yuntao Li

**Affiliations:** 1 Breast Center, The Fourth Hospital of Hebei Medical University, Shijiazhuang 050000, Hebei, China; 2 Animal Center, The Fourth Hospital of Hebei Medical University, Shijiazhuang 050000, Hebei, China

**Keywords:** miRNA-30d-5p, SERPINE1, fatty acid beta oxidation, breast cancer proliferation

## Abstract

Breast cancer (BC) is among the top three most prevalent cancers across the world, especially in women, and its pathogenesis is still unknown. Fatty acid β-oxidation is highly associated with breast cancer. Serpin family E member 1 (SERPINE1)-induced down-regulation of fatty acid β-oxidation can facilitate BC cell proliferation, invasion, and metastasis. In this paper, the difference of miR-30d-5p expressions in both cancerous tissues and para-carcinoma tissues was first detected. Next, the expressions of SERPINE1, long-chain acyl-CoA dehydrogenase (LCAD) and medium-chain acyl-CoA dehydrogenase (MCAD) in the aforementioned tissues were analyzed. Finally, miR-30d-5p mimics were supplemented to breast cancer cells to observe the miR-30d-5p effect upon breast cancer cells. Via immunofluorescence assay and Western blotting, it was found that cancerous tissues had lower expressions of miR-30d-5p, MCAD and LCAD and a higher expression of SERPINE1 than para-carcinoma tissues. The miR-30d-5p mimic group had a decreased SERPINE1 expression and increased MCAD and LCAD expressions compared with the NC group, thus inhibiting BC cell proliferation, invasion, and metastasis. To sum up, miR-30d-5p blocks the cell proliferation, invasion and metastasis by targeting SERPINE1 and promoting fatty acid β-oxidation. Preclinical studies are further required to establish a fatty acid β-oxidation-targeting therapy for breast cancer.

## INTRODUCTION

Breast cancer (BC) is among the top three most prevalent cancers worldwide, especially in females [1]. It is estimated that there exist approximately 2,088,849 new BC cases all over the world each year, occupying nearly a quarter of cancer cases in women [2]. As the relevant theories and technologies develop, a significant progress has been made in cancer therapy. However, the BC death rate still reaches about 30%. In 2018, the latest data from IARC showed that breast cancer affects 24.2% of women globally, 52.9% of whom are in the developing country [3]. In some developing countries, such as South America, Africa and Asia, breast cancer has already been a leading cause of cancer death, partly due to a lack of access to advanced diagnoses and treatment [4].

The mammary gland is an accessory gland of the skin and is a double-tube alveolar gland. The breast is composed principally of connective tissue and adipose tissue (AT). AT consists of numerous clusters of fat cells that are divided into lobules via thin and loose connective tissue layers. Based on the structure and function of adipose cells, adipose tissue is classified into yellow (white) AT and brown At, the former of which is the main component of the breast. Fatty acid plays a pivotal part in all stages of cancer [5, 6]. Normally, large amounts of fatty acids are required for rapidly proliferating cells, so that membrane synthesis can be facilitated and phospholipids are formed to support replication [7]. Targeting fatty acid metabolic reprogramming of tumor cells has gradually become the focus of research [8].

The serpin family E member 1 (SERPINE1) protein is a serine protease inhibitor E1. SERPINE1 becomes the dominating inhibitors of tissue plasminogen activator (tPA) and urokinase (uPA). Besides, it is the plasminogen activator and fibrinolysis activator [9]. It existed in platelets, plasma, endothelial, fibrosarcoma cells and hepatoma cells. Serine protease plays an indispensable part in signal transduction, cell adhesion, and cell metastasis [10–12]. By regulating tPA and uPA that convert proenzyme plasminogen to plasmin, SERPINE1 also activates matrix metalloproteinases and extracellular matrix degradation, thus promoting cell invasion [11]. It is verified that high SERPINE1 levels are linked to BC adverse outcomes [10].

MicroRNAs (miRNAs) function as negative gene expression regulators and get involved in the pathogenesis of multiple myeloma and other cancers, which may become a novel therapeutic approach. miR-30s has a lower expression in multiple myeloma samples than in normal plasma cells. miR-30d-5p serves as one kind of tumor suppressor and gets involved in tumor development [13–15]. Nevertheless, the miR-30d-5p function in breast cancer has never been reported.

It was discovered in this research that highly-expressed miR-30d-5p was capable of decreasing the SERPINE1 gene expression, promoting fatty acid β-oxidation, and increasing LCAD and MCAD expressions, thus suppressing BC cell proliferation, invasion and metastasis.

## MATERIALS AND METHODS

### Gene expression and clinical data in The Cancer Genome Atlas (TCGA) database

In terms of BC, miRNA data and mRNA data (RNA-Seq v2) were obtained from TCGA. Besides, clinical data were supplied by cBioPortal database used for clinical analyses of the malignant phenotype. The differential expression between cancerous tissues and para-carcinoma tissues was analyzed using DESeq2. With a false discovery rate (FDR) <0.05 and a fold change >2, the genes were considered to be differential expression.

### Gene set enrichment analysis (GSEA)

Apart from fat metabolism, the correlation between miR-30d-5p/SERPINE1 expression and cell proliferation, invasion, metastasis was analyzed using GSEA v2.2 software. The Molecular Signatures Database offered the total gene sets. Significance thresholds were measured using permutation analytics (1,000 permutations). While the FDR score was <0.25, a gene set was prominently enriched.

### Cell culture and transfection

BC MCF7 cells were fostered in high-glucose DMEM (Abcam, Shanghai, China) added with 100 U/mL penicillin, 100 mg/L streptomycin, and 10% FBS (Abcam, Shanghai, China) under 5% CO_2_, floating in serum-free DMEM/F12 culture medium (Abcam) with 20 ng/mL EGF, 20 ng/mL b-FGF, and 2% B27 (Abcam). Then, we spent 10 days in culturing them within ultra-low attachment plates. miR-30d-5p mimics were synthesized within the GenePharma company (Shanghai, China). Additionally, they were transfected into cells using Lipofectamine 2000 reagent in Invitrogen (USA) in light of the methods of Liu, et al. [16].

### Nude mouse tumor-bearing model

In total, 16 BALB/c-nu nude mice aged 6 weeks were supplied by Henan SCBS Biotechnology Co., Ltd. (China). The needles were inserted into the upper part of the waist of the nude mice, without piercing the skin or the muscle layer, and MCF7 cells pre-stored in an ice box were inoculated at a dose of 1×10^7^/200 μL per mouse within 1 h. The distance from the inoculation site was smaller than the length of the needle. For this research, we conducted the experiments on animals. After successful tumorigenesis, the tumor volume was detected via hematoxylin-eosin (HE) staining. Besides, cancerous tissues and para-carcinoma tissues were harvested for later assays.

### HE staining

The tumor tissue sections were deparaffinized, soaked in distilled water and stained with hematoxylin for several minutes, followed by color separation in acid and ammonia each for a few seconds. After rinsing with running water for 1 h, we added distilled water to those sections, spent 10 minutes in dehydrating them with alcohol and spent 3 minutes in staining them with eosin. Then, we dehydrated those section with pure alcohol and transparentizing them with xylene. Ultimately, they were added with neutral gum, and investigated using an optical microscope.

### Dual-luciferase reporter assay

GenScript (Nanjing, China) constructed dual-luciferase reporter (DLR) plasmids (miR-30d-5p-wt and miR-30d-5p-mut). SERPINE1-wt was transfected into BC cells with miR-30d-5p NC. Simultaneously, SERPINE1-mut was transfected into BC cells with miR-30d-5p mimic. After 48 hours, we detected the luciferase activity according to manufacturer’s protocol.

### Immunofluorescence assay

Frozen tissues in OCT chemical compound were sliced into sections (30 μm) on one CM3000 cryostat, permeabilized into 0.3% Triton X-100/PBS solution and hatched using primary antibodies against miR-30d-5p, Ki-67 (1:200, Osaka, Japan), SERPINE1 (1:200, Santa Cruz, USA), LCAD (1:500, Shanghai, China), and LCAD (1:500, Shanghai, China) diluted within 0.03% Triton X-100/PBS using 10% normal goat serum during the night at the temperature of 4 degrees. The following day, those sections were hatched using Alexa-Fluor 488 and Alexa-Fluor 568 secondary antibodies (1:250-1:500), and the nuclei were stained using Hoechst 33258 (Nacalai Tesque, Kyoto, Japan). Photographs were taken using a microscopic system connected with one digital camera. Besides, they were processed by means of Image and Photoshop.

### Cell counting kit-8 (CCK-8) assay

MCF-7 cells with or without miR-30d-5p mimics were fostered within a 96-well plate at 2×10^3^ cells per well. As per production specification, the value of optical density (OD) was measured at 570 nm and tested using CCK-8 assay (Dojindo, Tokyo, Japan).

### Reverse transcription-polymerase chain reaction (RT-PCR) and Western blotting

We lysed MCF-7 cells using TRIzol reagent (Sigma, USA), with the aim of extracting all RNAs. Reverse transcription easily impacted them via PrimeScript RT Reagent Kit (Takara, Japan). Additionally, they were quantified via SYBR Green Labeling (Takara). The relative miR-30d-5p and mRNAs expressions were normalized to U6 and GAPDH, separately, and figured out by means of a 2^-ΔΔCT^ method [17]. The following are the primers used: miR-30d-5p F: 5’-CCTGTTGGTGCACTTCCTAC-3’, R: 5’-TGCAGTAGTTCTCCAGCTGC-3’; GAPDH F: 5’-GGTCTCCTCTGACTTCAACA-3’, R: 5′-GCCAAATTCGTTGTCATAC-3′.

We extracted the total protein from the cells via RIPA lysis buffer. Besides, the protein was quantified via the BCA Protein Quantification Kit. Next, the protein was electrophoresed within SDS-PAGE, and delivered onto a PVDF membrane. That membrane was impeded using 5% skim milk powder for 2 h, and hatched using primary antibodies during the night and using secondary antibodies the next day. Finally, the electrochemiluminescence (ECL) reagent was used for visualization. We independently repeated the assay 3 times.

### Transwell assay

The cells were prepared by single-cell suspensions and cultivated into the upper chamber (Corning, USA) covered with Matrigel (0.1%, Millipore, USA). After 48 h, those cells were immobilized with 4% paraformaldehyde for 10 min and stained with trypan blue. Using an optical microscope, we observed, counted and imaged those cells on the lower membrane surface.

### Statistical analysis

Pearson correlation analysis was implemented between miR-30d-5p and SERPINE1. Images were drawn by means of GraphPad Prism 6. The total data was expressed as mean ± standard deviation. Differences were contrasted between two groups via independent-samples *t*-tests. *P*-value was less than 0.01, which indicated the difference was statistically significant.

## RESULTS

### Expressions of miR-30d-5p in breast cancer tissues and para-carcinoma tissues

**Figure 1:**
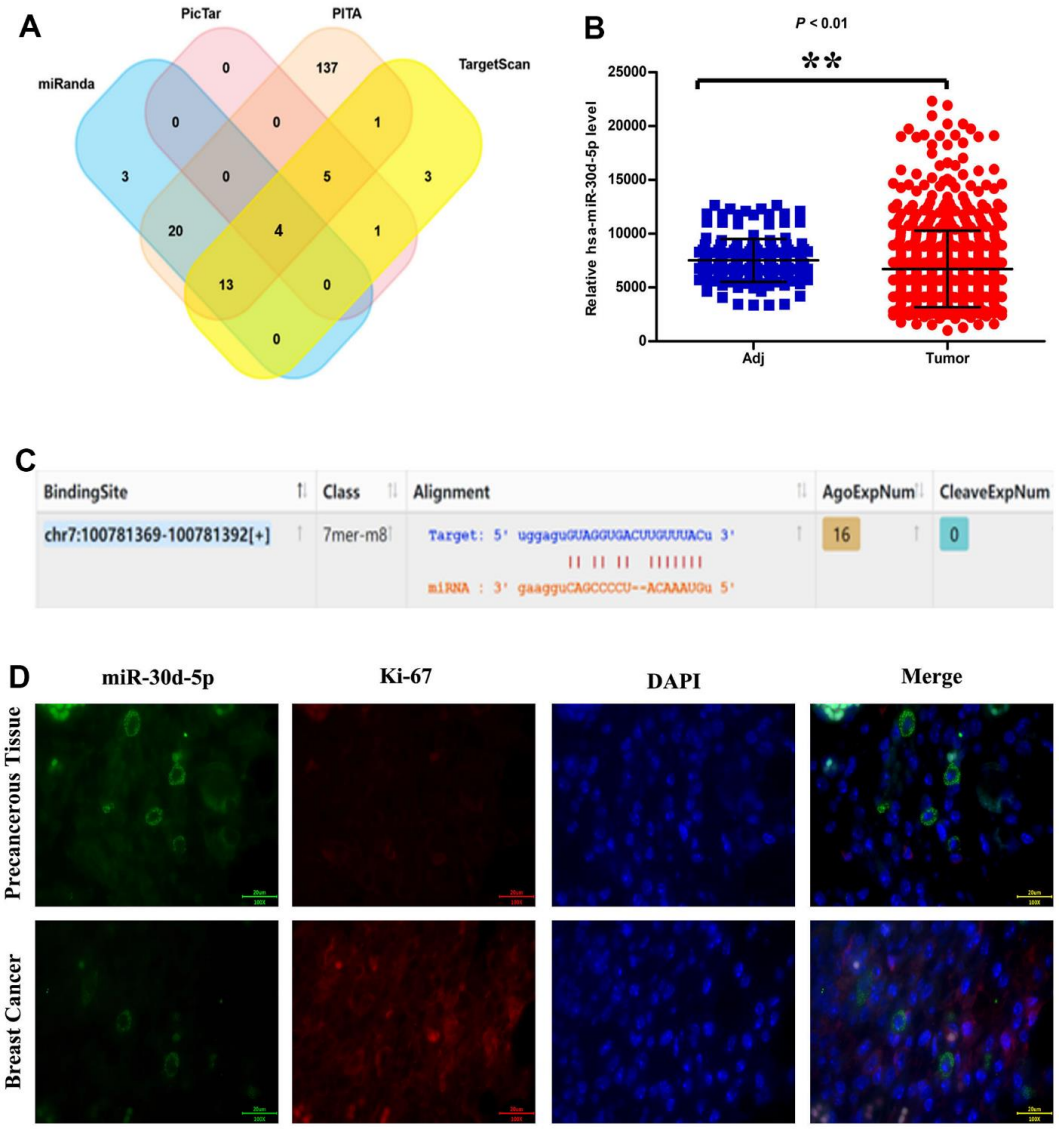
miRNA target prediction software: miRanda, PicTar, PITA and TargetScan (**A**). Relative has-miR-30d-5p expression level in adjacent tissue and tumor (**B**). miR-30d-5p information, including binding site, gene sequence and miRNA sequence (**C**). miR-30d-5p, Ki-67, DAPI Immunofluorescence staining in pericancerous tissue and breast cancer (**D**). Scale bar=20 μm. **, *P*<0.01.

SERPINE1 up-regulation in BC tissues was more significant than that in para-carcinoma tissues (Figure 1A). It was showed in GSEA results that the gene sets associated with cell proliferation, invasion and metastasis were prominently enriched in high miR-30d-5p expression group (Figure 1B). SERPINE1 was correlated positively with lymph node metastasis (Figure 1C). It was found by immunofluorescence assay on miR-30d-5p (green) and Ki67 (red) that the miR-30d-5p expression in BC tissues was lower than that in para-carcinoma tissues (Figure 1D).

### SERPINE1 was more highly expressed in tumor tissues than in para-carcinoma tissues

**Figure 2:**
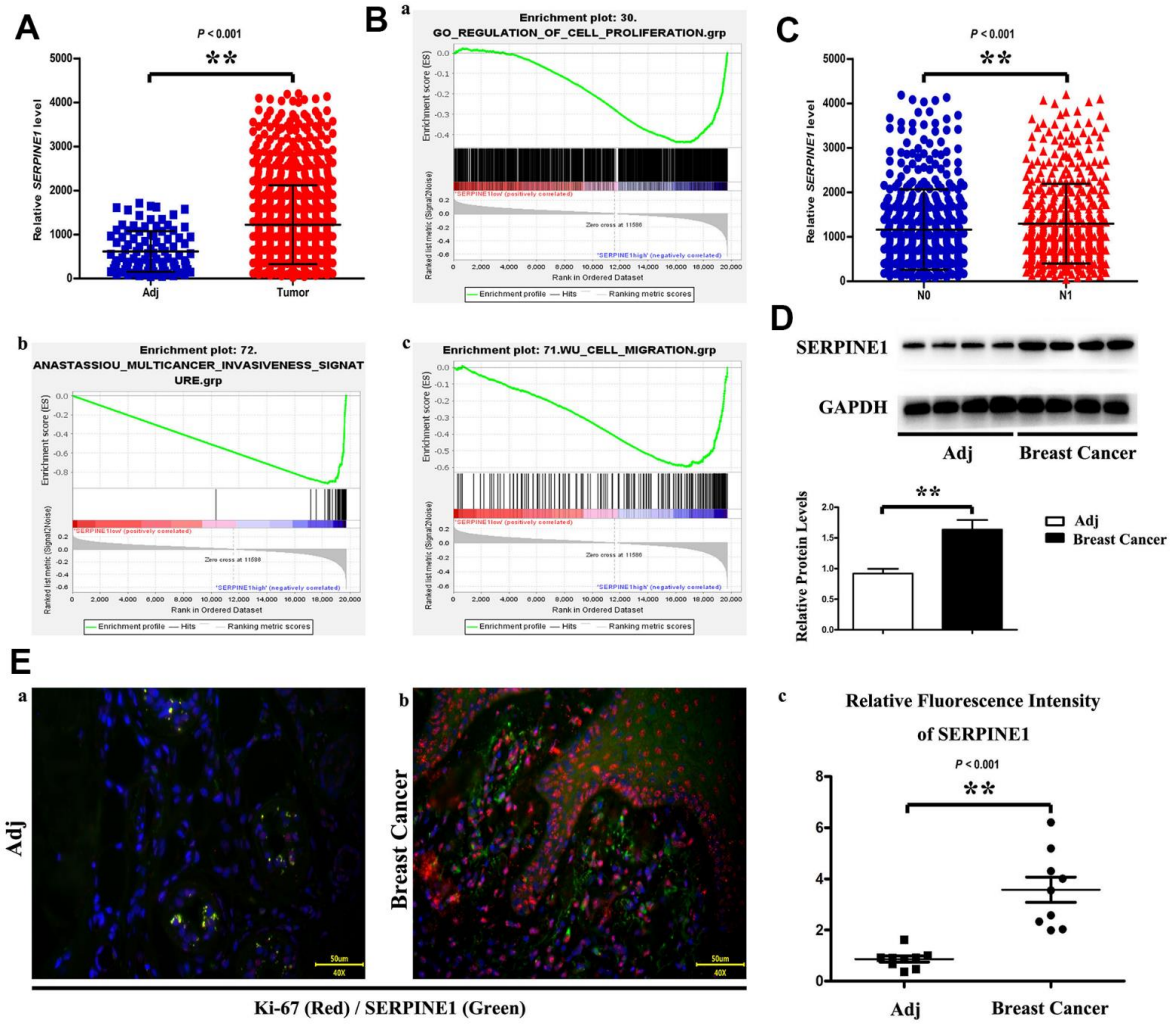
SERPINE1 expression level is higher in tumor than in adjacent tissue (**A**). Statistics showed proliferation, invasion and migration of breast cancer cell increase as the SERPINE1 expression increasing (**B**). PCR data demonstrate SERPINE1 level is significantly different between N0 and N1 (**C**). Western blot shows that SERPINE1 expressed higher level in tumor than in adjacent tissue (**D**). Ki-67 and SERPINE1 immunofluorescence staining in adjacent tissue and breast cancer (**a**, **b**); fluorescence intensity of SERPINE1 is expressed higher in breast cancer (**c**) (**E**). Scale bar=50 μm. **, *P*<0.01.

miRNAs able to bind to SERPINE1 were prognosticated with TargetScan, miRanda, PicTar and PITA databases (Figure 2A). The miR-30d-5p and SERPINE1 binding points are illustrated in Figure 2B. The four co-targeted miRNAs were analyzed. Additionally, it was discovered that only miR-30d-5p down-regulation in BC tissues was passively connected to SERPINE1 expression (Figure 2A, 2B). Next, GSEA results indicated that the low miR-30d-5p expression was associated with BC phenotype (Figure 2C). According to Western blotting, the protein SERPINE1 expression in cancerous tissues was high, in comparison with that in para-carcinoma tissues (Figure 2D), which was verified by immunofluorescence staining (Figure 2E-a/b/c).

### Fatty acid β-oxidation was regulated by SERPINE1 and miR-30d-5p

**Figure 3:**
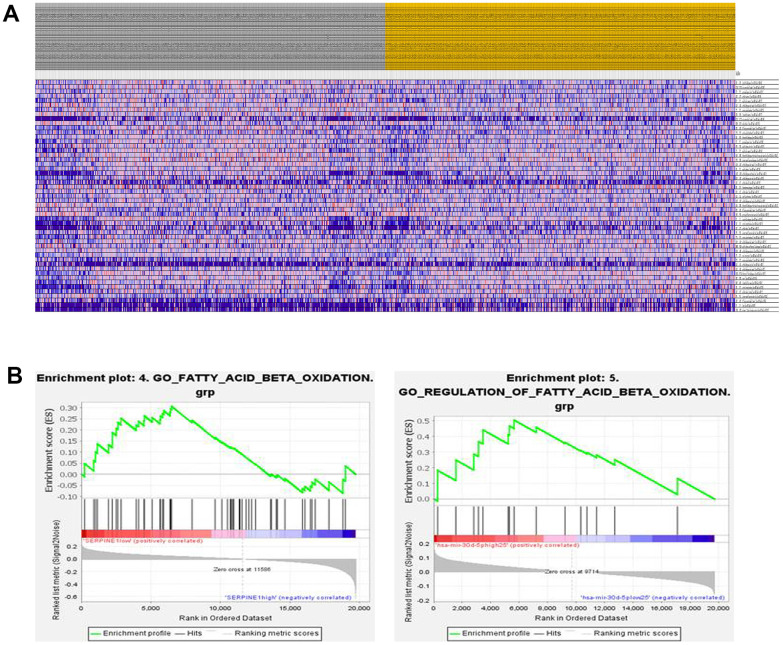
Heatmap shows the enrichment score of cell proliferation, migration, invasion and fat metabolism related gene set (**A**). GSEA (Gene Set Enrichment Analysis) enrichment plot. The plot shows the enrichment scores for fatty acid beta oxidation gene set tested in the analysis. The x-axis represents the ranked list of genes, while the y-axis represents the enrichment score. Left panel shows the plot ranked by SERPINE1 (down-regulation) and right panel shows the enrichment plot ranked by miR-30d-5p (up-regulation) (**B**).

According to the gene set enrichment analysis, it has been found that several pathways, including those related to cell proliferation, migration, invasion, and fat metabolism, are enriched with genes associated with SERPINE1 and miR-30d-5p (as shown in Figure 3A). Furthermore, it has been observed that the decreased expression of SERPINE1 leads to the stimulation of fatty acid β-oxidation (as depicted in the left panel of Figure 3B). For another, it has been shown that the increased miR-30d-5p expression stimulates fatty acid β-oxidation (as depicted in the right panel of Figure 3B).

### Expressions of MCAD and LCAD in breast cancer tissues and para-carcinoma tissues

**Figure 4:**
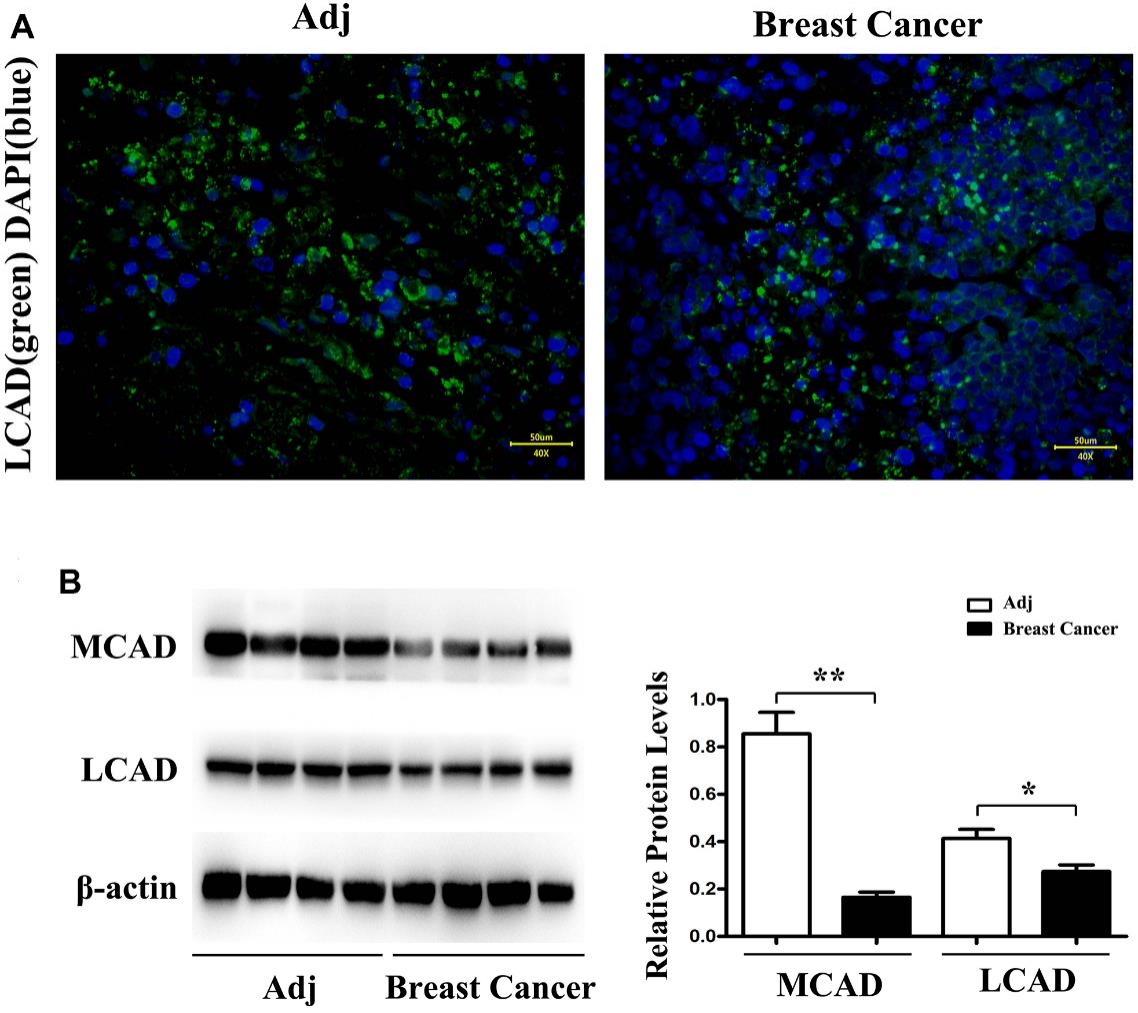
LCAD (long-chain Acyl-CoA dehydrogenase) (green) immunofluorescence staining in adjacent tissue and breast cancer (**A**). Western blotting demonstrates that MCAD (Medium-chain acyl-CoA dehydrogenase) and LCAD (long-chain Acyl-CoA dehydrogenase) expression level is significantly higher in adjacent tissue than in breast cancer (**B**). n = 32 (4 fields each from 8 mice). Scale bar=50 μm. **, *P*<0.01.

Using immunofluorescence staining, it was observed that the LCAD expression in para-carcinoma tissues was more elevated than that in BC tissues (Figure 4A). The Western blotting results uncovered that the MCAD and LCAD expressions in para-carcinoma tissues were prominently high, by comparison with those in BC tissues (Figure 4B).

### miR-30d-5p mimics could reduce tumor volume by specifically targeting SERPINE1

**Figure 5:**
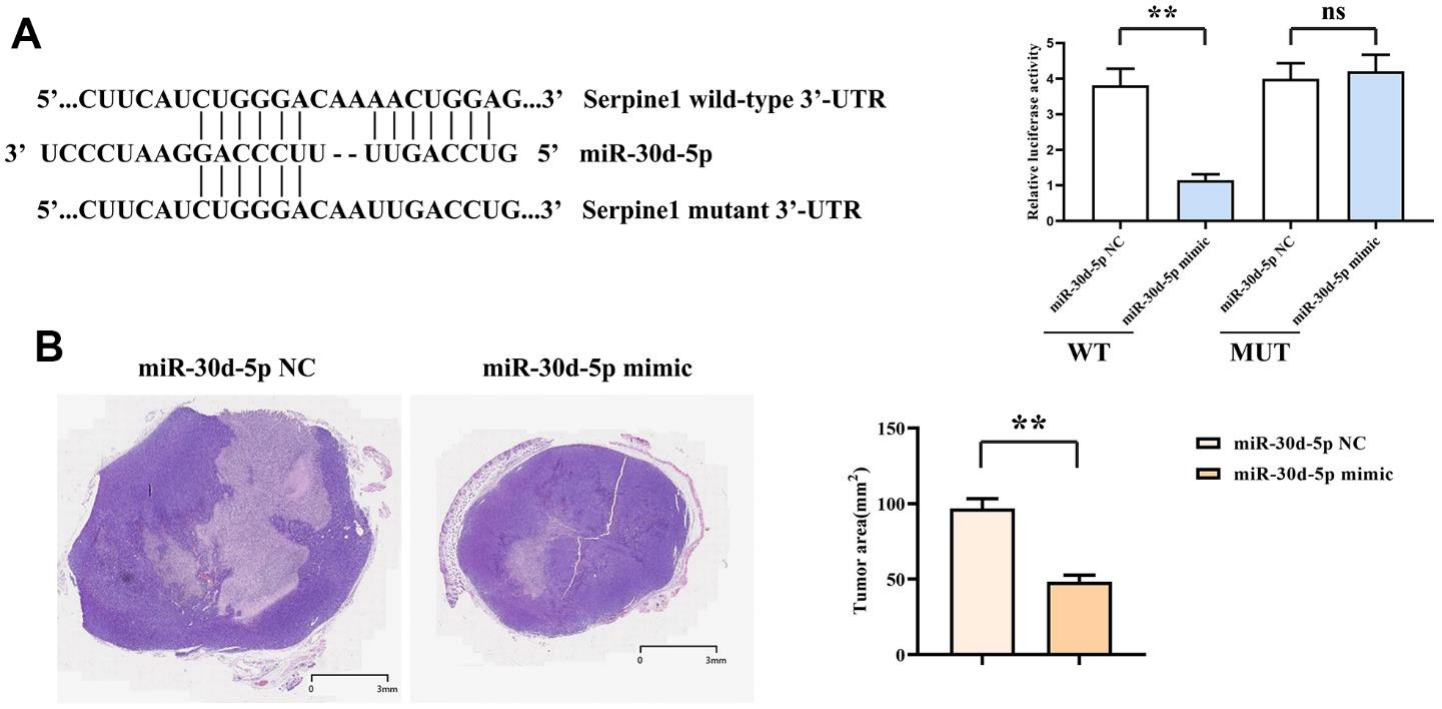
Dual-luciferase assay results (**A**). HE staining results and area statistics of nude mouse tumor sections (**B**). **, *P*<0.01; ns, *P*>0.05.

The related immunofluorescence information was exhibited in DLP assay. Of the transfected cells using SERPINE1-wt in the miR-30d-5p mimic group, the immunofluorescence intensity declined more significantly than that in the miR-30d-5p NC group. However, there was no dissimilarity in the immunofluorescence intensity of cells transfected with SERPINE1-mut between those two group (Figure 5A). In the nude mouse tumor-bearing experiment, it was discovered that the tumor volume in the mimic group decreased significantly by comparison with that in the NC group (Figure 5B).

### miR-30d-5p mimics inhibited migration, proliferation and invasion of breast cancer cells

**Figure 6:**
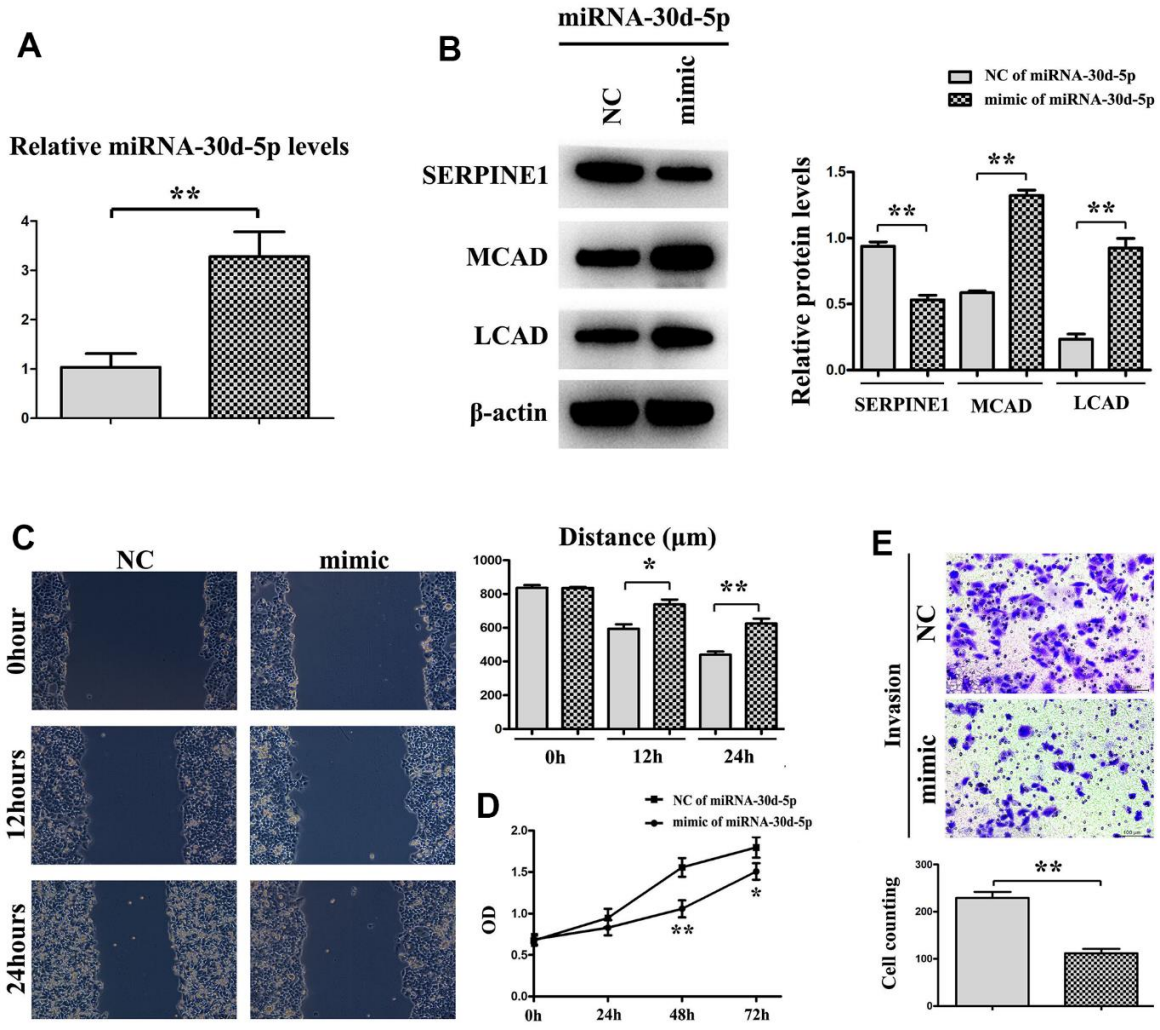
miRNA-30d-5p expression is higher in mimic than NC (**A**). Western blot shows that the expression of SERPINE1 is higher in NC than mimic, but MCAD (Medium-chain acyl-CoA dehydrogenase) and LCAD (long-chain Acyl-CoA dehydrogenase) are the opposite (**B**). Tumor migration experiment shows the distance of mimic group is shorter than NC group (**C**). The expression is significantly different in mimic group at 24h, 48h and 72h, compared with in NC group (**D**). Cell counting shows invasion is decreased in mimic than NC through trypan blue staining (**E**). **, *P*<0.01; *, *P*<0.05.

**Figure 7:**
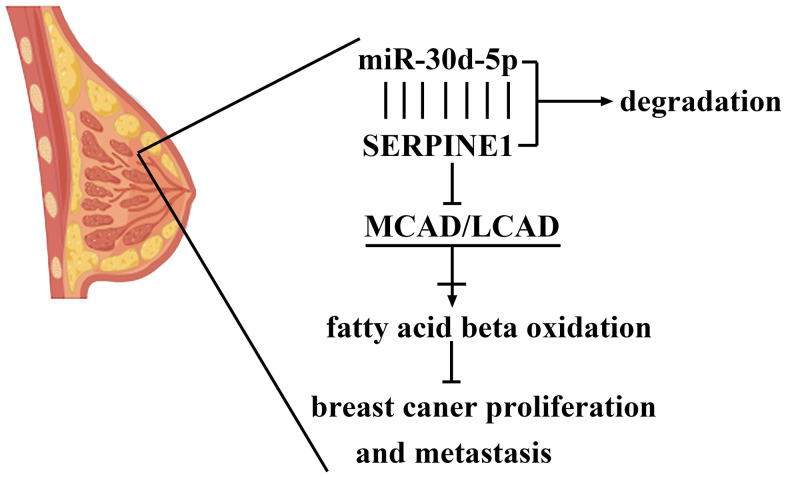
The mechanisms of miR-30d-5p in the modulation of axis of SERPINE1/MCAD/LCAD in breast cancer.

In Figure 6A, the miR-30d-5p level in that mimic group was high, by comparison with that in the NC group. Through Western blotting, it was shown that the SERPINE1 expression was higher in the NC group, whereas the MCAD and LCAD expressions were higher in the mimic group (Figure 6B). In the mimic group the quantity of metastasizing cells was smaller than that in the NC group (Figure 6C). Moreover, OD values at 48 h and 72 h in the mimic group were prominently less than those in the NC group (Figure 6D). The number of invading cells in the mimic group was smaller than that in the NC group (Figure 6E). To sum up, BC cell proliferation, invasion and metastasis were blocked by miR-30d-5p with SERPINE1 targeted and fatty acid β-oxidation promoted (Figure 7).

## DISCUSSION

Breast cancer (BC) is a prevalent form of cancer in females. Besides, it becomes the second dominating reason for cancer deaths. The BC pathogenesis is intricate and involves numerous factors [17, 18]. miRNAs play an irreplaceable part in BC development and prognosis. Since a single miRNA controls the regulation of multiple target genes, miRNAs possess multi-target and efficient regulatory effects. Therefore, to target miRNAs may be a novel therapeutic strategy for cancer [18].

Energy metabolic reprogramming is thought to be one of the hallmarks of cancer, and an increasing number of studies have considered fatty acid oxidation as an essential process that contributes to transformation and tumorigenesis [19]. HIF-1 inhibits MCAD and LCAD, resulting in decreased levels of reactive oxygen species and enhanced proliferation. At the same time, a decreased LCAD expression can predict mortality [20]. According to many researches, it has been shown that miR-30d-5p gets involved in tumor development as a tumor suppressor in gallbladder cancer, prostate cancer, renal cell carcinoma, etc. In accordance with this research, miR-30d-5p expression in BC tissues was lower than that in para-carcinoma tissues. Likewise, MCAD and LCAD proteins, which are important intermediates in the fatty acid β-oxidation, were also lowly expressed in BC tissues by comparison to para-carcinoma tissues, while SERPINE1, an important regulatory protein involved in extracellular matrix reorganization and cell adhesion, was highly expressed in BC tissues. Furthermore, miR-30d-5p mimics were transfected into BC cells, for the purpose of inducing the its expression. The outcomes manifested that miR-30d-5p could increase MCAD and LCAD expressions by inhibiting SERPINE1.

The influence of fatty acid β-oxidation on tumor growth, proliferation, migration and invasion was explored in this study, but there are several other forms of fatty acid oxidation, such as fatty acid α-oxidation. SERPINE1 can regulate endothelial cell senescence and dysfunction induced by tumor necrosis factor [21, 22]. It was found by Western blotting that the relative protein expressions of MCAD and LCAD increased significantly with the decrease in SERPINE1. In addition, the wound healing assay indicated that the SERPINE1 decrease restrained cancer cell metastasis capacity, and the CCK-8 assay and monoclonal proliferation assay uncovered that cancer cell proliferation capacity was weakened as SERPINE1 decreased. Moreover, a DLR assay was designed, with a view to verifying that miR-30d-5p was capable of specifically targeting SERPINE1.

This study only explored the effect of fatty acid β-oxidation on the tumor at the cellular level. However, animal experiments were not performed, and the effect of antioxidant drugs upon cancer growth was not investigated. In future, we will focus on the influences of other forms of fatty acid oxidation on tumor growth and the influences of antioxidant drugs on tumor growth.
